# TAS‐115 inhibits PDGFRα/AXL/FLT‐3 signaling and suppresses lung metastasis of osteosarcoma

**DOI:** 10.1002/2211-5463.12827

**Published:** 2020-03-30

**Authors:** Naohiro Yasuda, Satoshi Takenaka, Sho Nakai, Takaaki Nakai, Shutaro Yamada, Yoshinori Imura, Hidetatsu Outani, Kenichiro Hamada, Hideki Yoshikawa, Norifumi Naka

**Affiliations:** ^1^ Department of Orthopaedic Surgery Osaka University Graduate School of Medicine Suita Japan; ^2^ Department of Orthopaedic Surgery Kawachi General Hospital Higashiosaka Japan; ^3^ Department of Orthopaedic Surgery Yao Municipal Hospital Japan; ^4^ Musculoskeletal Oncology Service Osaka International Cancer Institute Japan; ^5^ Department of Orthopaedic Surgery Toyonaka Municipal Hospital Japan

**Keywords:** LM8, lung metastasis, molecular targeted therapy, osteosarcoma, TAS‐115

## Abstract

Osteosarcoma is the most common malignant bone tumor in adolescence and childhood. Metastatic osteosarcoma has a poor prognosis with an overall 5‐year survival rate of approximately 20%. TAS‐115 is a novel multiple receptor tyrosine kinase inhibitor that is currently undergoing clinical trials. Using the mouse highly lung‐metastatic osteosarcoma cell line, LM8, we showed that TAS‐115 suppressed the growth of subcutaneous grafted tumor and lung metastasis of osteosarcoma at least partially through the inhibition of platelet‐derived growth factor receptor alpha, AXL, and Fms‐like tyrosine kinase 3 phosphorylation. We also show that these signaling pathways are activated in various human osteosarcoma cell lines and are involved in proliferation. Our results suggest that TAS‐115 may have potential for development into a novel treatment for metastatic osteosarcoma.

## Graphical abstract

TAS‐115 suppressed the growth of subcutaneous grafted tumor and lung metastasis of a mouse spontaneous highly metastatic osteosarcoma (OS) cell line, LM8. TAS‐115 exerts antitumor effects against OS cell lines partially via the inhibition of PDGFRα, AXL, and FLT‐3 signaling. TAS‐115 may be a potential novel therapeutic agent for patients with metastatic OS.

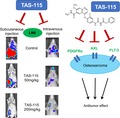

**Abbreviations**
FLT‐3: Fms‐like tyrosine kinase 3
OS: osteosarcoma
PDGFRs: platelet‐derived growth factor receptors
RTKs: receptor tyrosine kinases
TAM: Tyro3/AXL/Mer
VEGFRs: vascular endothelial growth factor receptors

Osteosarcoma (OS) is the most common malignant bone tumor in adolescence and childhood. Despite combined treatment with wide resection and chemotherapy, lung metastases occur in 40–50% of patients with OS [1, 2]. The survival of patients with metastatic or relapsed osteosarcoma has remained virtually unchanged over the past 30 years, with an overall 5‐year survival rate of 20–30% [2, 3, 4]. Therefore, new therapeutic options are required to improve prognoses for those patients. TAS‐115 is a novel inhibitor of multiple receptor tyrosine kinases (RTKs) that has been shown to inhibit c‐MET, vascular endothelial growth factor receptors (VEGFRs), platelet‐derived growth factor receptors (PDGFRs), and Tyro3/AXL/Mer (TAM) [5]. Recently, it was reported that TAS‐115 has a favorable tolerability profile and exhibits antitumor activity in human gastric cancer [5, 6], human lung cancer [7, 8], and synovial sarcoma [9]. Specifically, TAS‐115 has been reported to be effective against bone metastasis, derived from carcinomas [10, 11]. However, the efficacy of this drug for the treatment of OS remains to be elucidated. Here, we aimed to investigate the therapeutic potential of TAS‐115 *in vitro* in different OS cell lines and *in vivo* in a subcutaneous tumor and lung metastasis mouse model.

## Materials and methods

### Cell lines

We utilized the syngeneic mouse, highly metastatic OS cell line LM8. LM8 was established from the Dunn OS cell line by eight repeated intravenous injections of the cells derived from the lung nodule [12]. We used a clone of LM8 cells that stably expresses luciferase to evaluate lung metastasis using an *in vivo* imaging system (IVIS), which was kindly provided by Yui. The human Yamato‐SS cell line, derived from synovial sarcoma, was established from surgically resected tumors in our laboratory, as described previously [13].

LM8 luc, Yamato‐SS, human osteosarcoma cell lines (HOS, MG63, 143B, U2OS, and SaOS2), MC3T3‐E1 (murine osteoblastic cells), and NHDF (normal human dermal fibroblasts—adult) were maintained in Dulbecco’s modified Eagle’s medium (Nacalai Tesque, Tokyo, Japan) containing 10% heat‐inactivated FBS (Sigma‐Aldrich, St. Louis, MO, USA) at 37 °C in a humidified atmosphere of 5% CO_2_. LM8 luc, Yamato‐SS, and human OS cell lines were authenticated by short tandem repeat analysis.

### Reagents and antibodies

TAS‐115 [4‐[2‐fluoro‐4‐[[[(2‐phenylacetyl)amino]thioxomethyl]amino]‐phenoxy]‐7‐methoxy‐N‐methyl‐6‐quinolinecarboxamide] was provided by Taiho Pharmaceutical Co., Ltd. (Tsukuba, Japan). CP‐673451 (PDGFR inhibitor), TP‐0903 (AXL inhibitor), and quizartinib [Fms‐like tyrosine kinase 3 (FLT‐3) inhibitor] were purchased from Selleck Chemicals (Houston, TX, USA). According to the manufacturer’s instructions, TAS‐115, CP‐673451, TP‐0903, and quizartinib were prepared in dimethyl sulfoxide (DMSO; Sigma‐Aldrich), before their addition to the cell cultures for the *in vitro* experiments. TAS‐115 was diluted in water with 2‐hydroxypropyl‐β‐cyclodextrin (HPβCD) to the appropriate concentrations for the *in vivo* experiments, according to the manufacturer’s instructions. Antibodies against platelet‐derived growth factor receptor alpha (PDGFRα) (#3174), p‐PDGFRα (Tyr^849^; #3170), p‐AXL ( #5724), AXL ( #8661), FLT‐3 (#3462), p‐FLT‐3 (#3461), poly(ADP‐ribose) polymerase (PARP) (#9542), CD31 (PECAM‐1) (#77699), and β‐actin (#4970), and anti‐rabbit IgG horseradish peroxidase‐linked secondary antibody (#7074) were purchased from Cell Signaling Technology, Inc. (Danvers, MA, USA). The antibody against AXL (ab32828) was purchased from Abcam (Cambridge, UK).

### Immunoblot analysis

For the lysate preparation, cells were first washed with phosphate‐buffered saline and lysed in radioimmunoprecipitation assay buffer (Thermo Scientific, Waltham, MA, USA) supplemented with 1% protease/phosphatase inhibitor cocktail (Cell Signaling Technology). Protein concentrations were determined using the bicinchoninic acid method (Thermo Scientific). The cell proteins were separated on 4–12 % Bis‐Tris gels (Life Technologies, Carlsbad, CA, USA) and transferred to polyvinylidene difluoride membranes (Nippon Genetics, Tokyo, Japan). The membranes were blocked at room temperature in Tris‐buffered saline, containing 5% skim milk and Tween‐20 (TBS‐T). Then, the membranes were incubated overnight with primary antibodies in Can Get Signal solution 1 (Toyobo Life Science, Tokyo, Japan) at 4 °C, followed by incubation with the secondary antibodies in Can Get Signal solution 2 (Toyobo Life Science) for 1 h at room temperature. After washing with TBS‐T, immunoreactive bands were visualized using enhanced chemiluminescent reagents [ECL Prime (GE Healthcare Life Sciences) and ImmunoStar LD (Wako, Osaka, Japan)].

### Cell proliferation and viability assay

The cell proliferation rate was measured using the premixed WST‐1 cell proliferation assay system (Takara Bio, Inc., Otsu, Japan). LM8 and Yamato‐SS cells were seeded in 96‐well plates at 2000 cells/well and treated 24 h after seeding with various concentrations of TAS‐115 (0–100 μm) for 48 h. The relative cell proliferation rate was calculated by subtracting absorbance measurements obtained from a microplate reader at 690 nm from those obtained at 450 nm. To evaluate the proliferation of OS cells, 1 × 10^6^ (HOS, MG63, 143B, and LM8) and 2 × 10^6^ (SaOS2 and U2OS) cells were seeded in 6‐well plates and allowed to attach overnight. The next day, the cells were treated with 0, 0.1, 1, and 10 μm of TAS‐115. The number of cells was assessed using a Countess automated cell counter (Invitrogen, Carlsbad, CA, USA). The experiment was repeated three times for each OS cell line.

### Cell cycle analysis

LM8 cells (1 × 10^6^ per dish) were seeded in 10‐cm culture dishes, incubated overnight, and then treated with TAS‐115 or vehicle (DMSO) for 24 h. The cells were harvested and stained with propidium iodide (PI) solution (25 μg·mL^−1^ PI, 0.03% NP‐40, 0.02 mg·mL^−1^ RNase A, and 0.1% sodium citrate) for 30 min at room temperature. For cell cycle analysis, we used the BD FACSVerse™ flow cytometer (BD Biosciences, Franklin Lakes, NJ, USA) for fluorescence‐activated cell sorting (FACS) according to the manufacturer’s protocol.

### *In vivo* syngeneic mouse model

The animal studies were performed in accordance with a guideline approved by the Institutional Animal Care and Use Committee of the Osaka University Graduate School of Medicine (27‐094‐006, 28‐015‐004). In the subcutaneous inoculation model, LM8 cells (1 × 10^7^) were inoculated subcutaneously into the flank of 5‐week‐old female C3H mice. One week after inoculation, TAS‐115 was orally administered once daily at a dose of 50 or 200 mg·kg^−1^ for 4 weeks. Lung metastasis and subcutaneous tumors were evaluated using IVIS 3 and 4 weeks after inoculation with LM8. Four weeks after inoculation, the mice were euthanized and the tumor weight was measured. Tumor volume (mm^3^) was defined as (*A* × *B* ^2^)/2, where *A* is the longest and *B* is the shortest diameter of the tumor. Tumor volume and mouse body weight were measured twice a week. In the intravenous injection model, LM8 cells (1 × 10^6^) were injected into the lateral tail vein of 5‐week‐old female C3H mice. One week after LM8 injection, TAS‐115 was orally administered once daily at a dose of 50 or 200 mg·kg^−1^ for 3 weeks. Lung metastases were evaluated using IVIS at 2 and 3 weeks after injection of LM8. For immunoblot analyses of tumor tissue lysate, mice bearing tumors were orally treated with TAS‐115 (200 mg·kg^−1^) for three consecutive days. Three hours after the last drug administration, the tumors were resected and extracted in Tissue Protein Extraction Reagent (T‐PER; Thermo Scientific) supplemented with 1% protease/phosphatase inhibitor cocktail. The doses of TAS‐115 were selected based on previous reports in which the daily administration of TAS‐115 (50–200 mg·kg^−1^) resulted in significant growth inhibition of the tumors [5, 6, 9, 11].

### *In vivo* imaging

Mice received d‐luciferin (Caliper Life Sciences, Waltham, MA, USA) at a dose of 100 μg per mouse by intraperitoneal injection 15 min prior to imaging. A region of interest (ROI) of the same size and shape, covering the entire thoracic cavity, was applied to every image. Total flux in the ROI was measured. Data were analyzed by using IVIS Living Image software (Caliper Life Sciences).

### Immunohistochemistry

Excised tumors were fixed in 10% neutral‐buffered formalin and embedded in paraffin. Sections (4 μm) were deparaffinized and dehydrated. After antigen retrieval in 10 mm citrate buffer at 95 °C for 30 min, endogenous peroxidase activity was blocked with methanol containing 3% H_2_O_2_ for 10 min. The sections were incubated overnight with primary antibodies at 4 °C, followed by 1 h of incubation with secondary antibodies. Finally, labeled sections were stained with 3,3′‐diaminobenzidine tetrahydrochloride (DAB; Dako, Glostrup, Denmark) and counterstained with hematoxylin.

### Measurement of tumor vascular density

Immunohistological staining of anti‐CD31 antibody in LM8 tumors was performed for each treatment group. Pictures were taken of three fields/section of each specimen of each treatment group in the best‐stained tumor area (×200). The microvessel density (MVD, number of tumor vessels per mm^2^) was calculated using the equation below:

$$\text{MVD}=\frac{\text{total}\;\text{number}\;\text{of}\;\text{vessels}\;\text{in}\;\text{three}\;\text{visual}\;\text{fields}}{\text{area}\;\text{of}\;\text{one}\;\text{visual}\;\text{field}\times 3}$$

### Phospho‐RTK arrays

A mouse phospho‐RTK array kit (R&D Systems, Minneapolis, MN, USA) was used to measure the relative level of tyrosine phosphorylation of 39 distinct receptor kinases. LM8 cells were cultured with/without TAS‐115 for 24 h and lysed using lysis buffer. For collecting the lysate from the subcutaneous tumor, or lung nodule in mice, a piece of the tumor sample was minced and homogenized in T‐PER supplemented with 1% protease/phosphatase inhibitor cocktail on ice immediately after tumor resection. The arrays were blocked with blocking buffer and incubated with 300 μg of cell lysate overnight at 4 °C. The arrays were washed, incubated with a horseradish peroxidase‐conjugated phosphotyrosine detection antibody, treated with chemiluminescent, and exposed to film.

### Statistical analysis

We used Student’s *t*‐tests to determine the significance between the untreated controls and the various treatments in the *in vitro* and *in vivo* experiments. Values of *P* < 0.05 were statistically significant.

## Results

### TAS‐115 suppresses the proliferation of LM8 cells *in vitro*

**Fig. 1:**
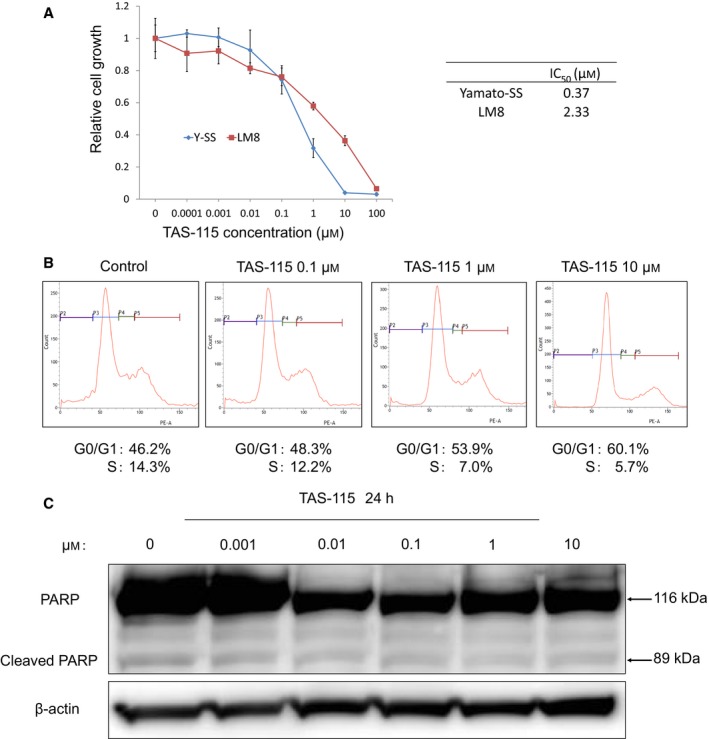
TAS‐115 inhibits the growth of LM8 cells by inducing G0/G1 cell cycle arrest and does not induce apoptosis. (A) LM8 and Yamato‐SS cells (1 × 10^3^) were treated with 0 to 100 μm TAS‐115 for 48 h, and the relative cell proliferation rates were measured by the WST‐1 assay. Bars represent the SD. The calculated IC_50_ values of each cell line are shown. (B) The effect of TAS‐115 on the cell cycle. LM8 cells were treated with 0.1% DMSO (control) or 0.1–10 μm TAS‐115 for 24 h. After treatment, the cells were stained with PI and analyzed by flow cytometry. (C) The effect of TAS‐115 on PARP cleavage in LM8 cells. Cells were treated with 0.1% DMSO (control) or 0.001–10 μm of TAS‐115 for 24 h.

We performed the WST‐1 cell proliferation assay to examine the *in vitro* antitumor activity of TAS‐115 in LM8 cells. TAS‐115 dose‐dependently inhibited the proliferation of LM8 cells (Fig. 1A) and of the synovial sarcoma cell line Yamato‐SS, as was previously reported [9]. Flow cytometric analyses showed that TAS‐115 increased the percentage of cells in the G0/G1 phase and decreased the percentage of cells in the S phase in the LM8 cells in a dose‐dependent manner (Fig. 1B). Immunoblot analyses revealed that the levels of cleaved PARP were not elevated after 24‐h treatment with TAS‐115 (Fig. 1C). Furthermore, we investigated the effect of TAS‐115 on normal cell lines, MC3T3‐E1 and NHDF. FACS showed that TAS‐115 caused only a slight increase in G0/G1 phase in normal cells compared with LM8 (Fig. S1). These results suggest that TAS‐115 suppressed cell proliferation by inducing G0/G1 cell cycle arrest without apoptotic changes in the LM8 cells, whereas the toxicity of TAS‐115 in normal cells was less than that of LM8.

### TAS‐115 inhibits the growth of subcutaneous tumors and the incidence of lung metastasis

**Fig. 2:**
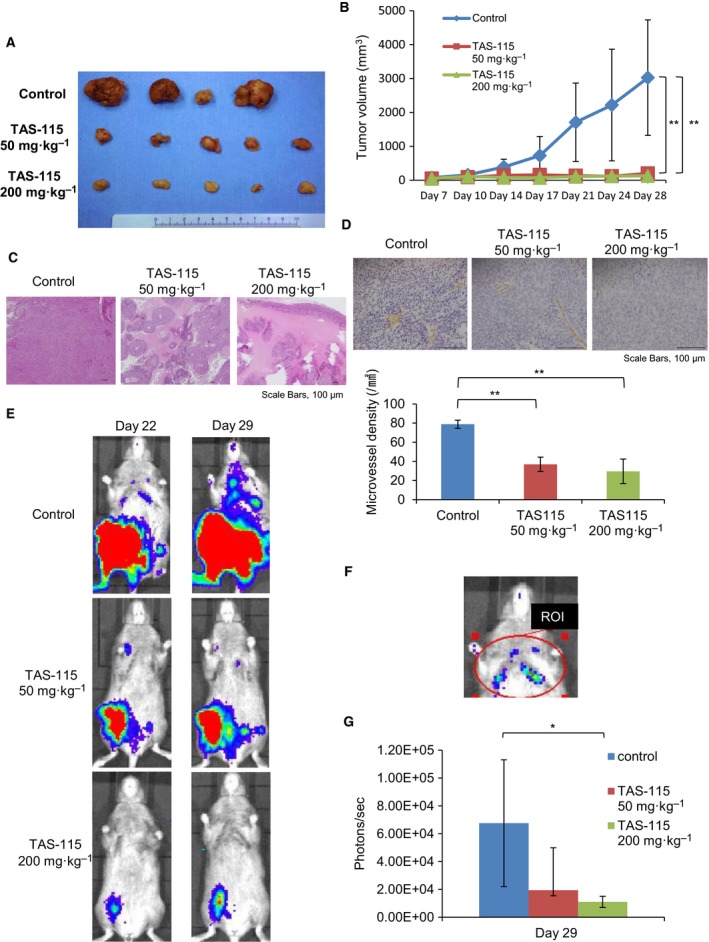
TAS‐115 strongly abrogates the growth of subcutaneous tumor and lung metastasis after subcutaneous injection of LM8. (A) The appearance of resected LM8 tumors at the end of the experiments. (B) Mice bearing LM8 were treated with 50 (*N* = 5) or 200 (*N* = 5) mg·kg^−1^ of TAS‐115, or the vehicle (control, *N* = 4). Bars represent the SE. **P < 0.01 in Student’s *t*‐test from control. (C) Histological images of LM8 tumors are shown (HE staining). Scale bars, 100 μm. (D) Immunohistological staining of CD31 in LM8 tumors for each treatment group (×200). Scale bars, 100 μm. Microvessel density (MVD) of LM8 tumors. Bars represent the SE. **P < 0.01 in Student’s *t*‐test from control. (E) The representative bioluminescence imaging *in vivo* of LM8 at 22 and 29 days after subcutaneous inoculation. Bioluminescence is presented as a pseudoscale: red, highest photon flux; and blue, lowest photon flux. (F) A region of interest (ROI) of the same size and shape, covering the entire thoracic cavity. (G) Quantification of bioluminescence imaging signal intensity in the three groups at 29 days after inoculation. Quantified values are shown in total flux. Bars represent the SE. *P < 0.05 in Student’s *t*‐test from control.

We investigated the growth of tumors after the subcutaneous injection of LM8. Mice bearing tumors were treated daily with an oral dose of 50 or 200 mg·kg^−1^ TAS‐115, or water with HPβCD as a control, 1 week after subcutaneous injection of LM8 cells. TAS‐115 (50 and 200 mg·kg^−1^) suppressed tumor growth comprising LM8 cells (Fig. 2A,B). Marked body weight loss was not observed in TAS‐115‐treated mice (Fig. S2). Histologically, a broad range of necroses were observed upon TAS‐115 treatment compared to the vehicle control (Fig. 2C). Immunohistochemical analyses revealed that TAS‐115 dose‐dependently decreased MVD (Fig. 2D), indicating that TAS‐115 has an anti‐angiogenesis effect. Using an IVIS 4 weeks after the inoculation of LM8 cells, TAS‐115 at a dose of 200 mg·kg^−1^ led to a decrease in bioluminescence intensity, not only in the subcutaneous tumor but also in the lung metastasis (Fig. 2E–G). These results suggest that TAS‐115 suppressed the growth of LM8 tumors and lung metastasis. However, it remains unclear whether TAS‐115 indirectly decreased the new lung metastasis formation by suppressing the growth of a subcutaneous tumor or directly suppressed the growth of a formed lung micro‐metastasis.

### TAS‐115 attenuates the growth of lung metastasis after intravenous injection of LM8

**Fig. 3:**
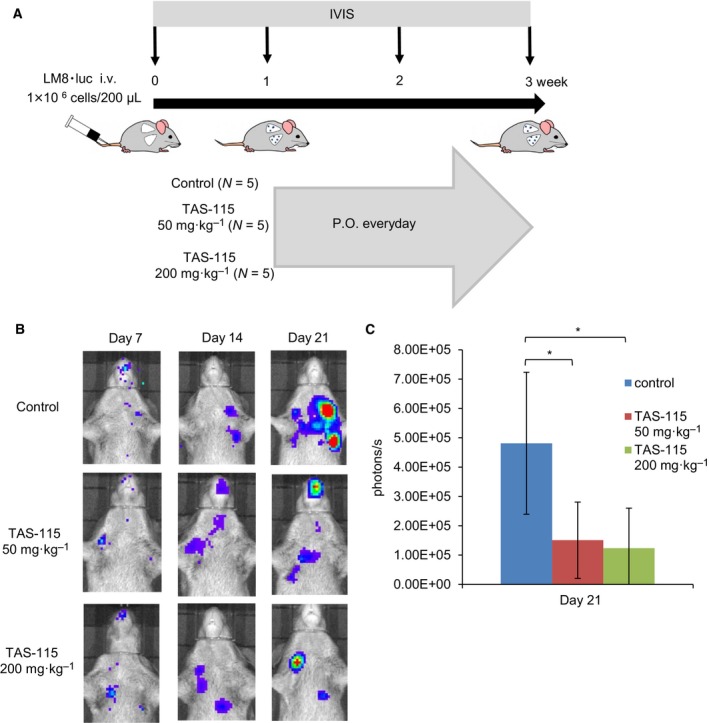
TAS‐115 inhibits the growth of lung metastasis after intravenous injection of LM8. (A) Schematic representation of the experimental setup after tail vein injection of LM8. Mice bearing LM8 were treated with 50 (*N* = 5) or 200 (*N* = 5) mg·kg^−1^ of TAS‐115, or the vehicle (control, *N* = 5). (B) The representative *in vivo* bioluminescence imaging of LM8 at 7, 14, and 21 days after intravenous injection. Bioluminescence is presented as a pseudoscale: red, highest photon flux; and blue, lowest photon flux. (C) Quantification of the bioluminescence imaging signal intensity in the three groups at 21 days after injection. Quantified values are shown in total flux. Bars represent the SE. *P < 0.05 in Student’s t‐test from control.

To investigate the growth inhibitory effect of TAS‐115 on a formed lung metastasis, we monitored lung metastasis after intravenous injection of LM8 cells by IVIS (Fig. 3A). One week after intravenous injection, bioluminescence signals from tumor cells were detected in the lung using IVIS (Fig. 3B) and the administration of TAS‐115 commenced. Three weeks after the injection of LM8 cells, TAS‐115 significantly decreased the intensities of bioluminescence in the lung compared to those of the controls (Fig. 3C). These results suggest that TAS‐115 suppressed the growth of a formed lung metastasis.

### Therapeutic potential of TAS‐115 via PDGFRα, AXL, and FLT‐3 signal inhibition for LM8

**Fig. 4:**
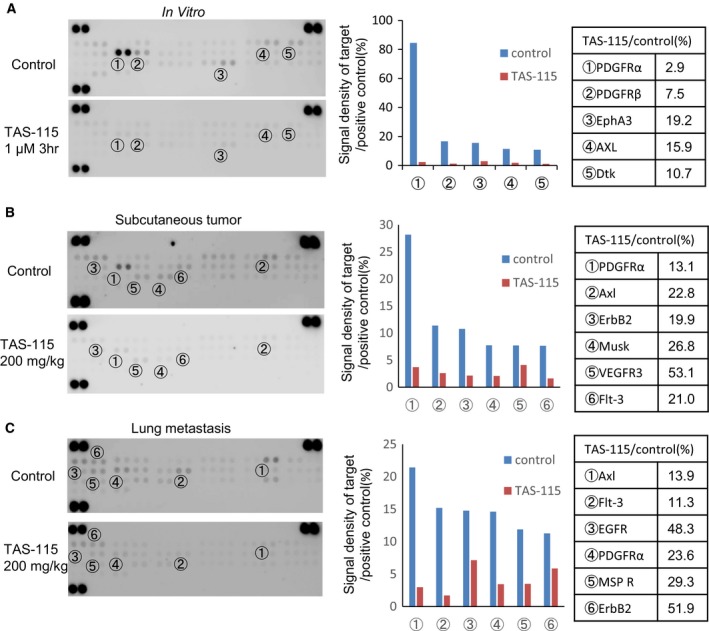
Revealing the differences in phosphorylation of the RTK against LM8. (A) RTK arrays for LM8 cells cultured *in vitro*. LM8 cells were treated with 10% FBS (control) or 1 μm of TAS‐115 for 3 h. (B) RTK arrays for LM8 cell lysate from subcutaneous tumor. Mice were administered TAS‐115 (200 mg·kg^−1^) or vehicle (control) orally for consecutive 3 days and euthanized 3 h after the final administration. (C) RTK arrays for LM8 cell lysate from lung metastasis. Mice were administered TAS‐115 (200 mg/kg) or vehicle (control) orally for consecutive 3 days and euthanized 3 h after the final administration.

**Fig. 5:**
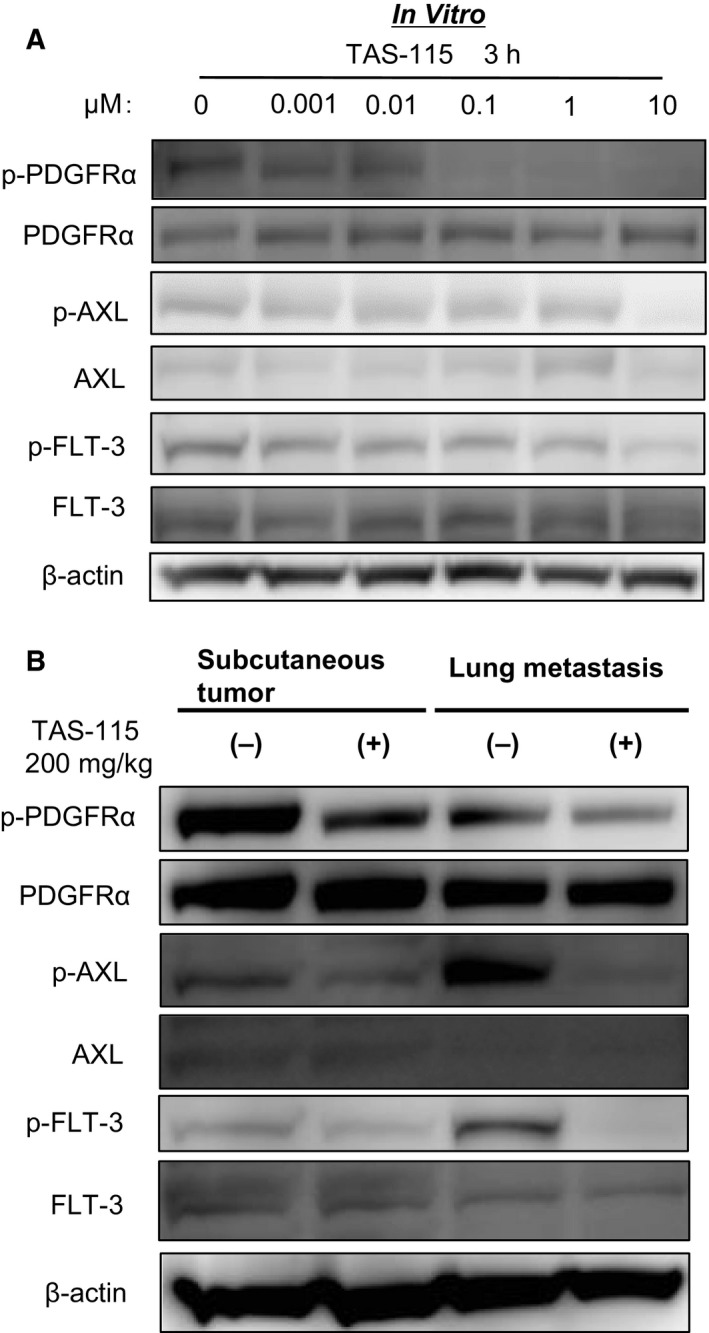
Inhibitory activities of TAS‐115 on PDGFRα, AXL, and FLT‐3 in vitro and in vivo. (A) LM8 cells were treated with 0.001–10 μm of TAS‐115 or 0.1% DMSO (control) for 3 h. (B) Mice bearing LM8 cells were treated with orally administered TAS‐115 (200 mg·kg^−1^) or control for consecutive 3 days and euthanized 3 h after the final administration.

Next, we investigated the mechanism by which TAS‐115 mediates growth inhibition of LM8 cells. The phospho‐RTK arrays revealed that PDGFRα demonstrated the highest levels of phosphorylation in the LM8 cell lysate (Fig. 4A). However, *in vivo*, not only PDGFRα but also some other TKs were phosphorylated. Notably, PDGFRα and AXL were strongly phosphorylated in the lysate of LM8 subcutaneous tumors (Fig. 4B), whereas AXL and FLT‐3 were in the lung metastasis lysate (Fig. 4C). These results suggest that the phosphorylation of RTKs differed between *in vitro* and *in vivo* conditions. Moreover, these phosphorylation levels of PDGFRα, AXL, and FLT‐3 were decreased in lysates treated with TAS‐115 *in vitro* and *in vivo.* We showed the effects of TAS‐115 on the PDGFRα, AXL, and FLT‐3 signaling pathways by immunoblot analyses *in vitro* and *in vivo*. PDGFRα phosphorylation was suppressed in LM8 cells after a 3‐h incubation with TAS‐115 at concentrations ranging from as low as 0.001 to 10 μm (Fig. 5A). To verify the inhibitory effects of TAS‐115 *in vivo*, we administered TAS‐115 (200 mg·kg^−1^) to mice bearing LM8 tumors. TAS‐115 at a dose of 200 mg·kg^−1^ inhibited the phosphorylation of PDGFRα, AXL, and FLT‐3 in both LM8 subcutaneous tumors and lung metastases (Fig. 5B). These results suggest that TAS‐115 inhibited LM8 tumor growth *in vitro* and *in vivo* through the inhibition of PDGFRα, AXL, and FLT‐3 signaling.

### PDGFRα, AXL, and FLT‐3 signaling are associated with the proliferation of several human OS cell lines

**Fig. 6:**
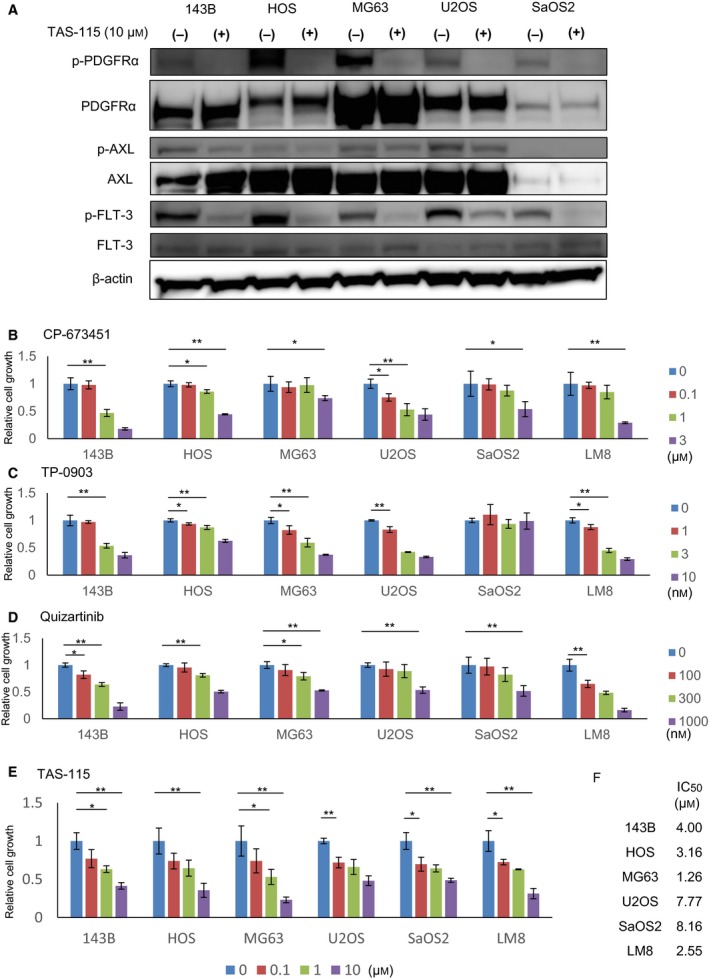
PDGFRα, AXL, and FLT‐3 signals are crucial for the proliferation of OS cell lines. (A) Phosphorylation status of RTKs in five human OS cell lines with or without TAS‐115. (B) SaOS2 (3 × 10^3^, 48 h), U2OS (2 × 10^3^, 72 h), 143B (1 × 10^3^, 72 h), HOS (2 × 10^3^, 72 h), MG63 (2 × 10^3^, 72 h), and LM8 (1 × 10^3^, 72 h) were treated with a PDGFR inhibitor at concentrations, ranging from 0 to 3 μm (*N* = 3 per group). The relative cell proliferation rates were determined using the WST‐1 assay. Bars represent the SD. *P < 0.05 and **P < 0.01 in Student’s t‐test. (C) SaOS2 (3 × 10^3^, 72 h), U2OS (2 × 10^3^, 72 h), 143B (1 × 10^3^, 72 h), HOS (2 × 10^3^, 72 h), MG63 (2 × 10^3^, 72 h), and LM8 (1 × 10^3^, 72 h) were treated with the AXL inhibitor at concentrations ranging from 0 to 10 nm (*N* = 3 per group). The relative cell proliferation rates were determined using the WST‐1 assay. Bars represent the SD. *P < 0.05 and **P < 0.01 in Student’s *t*‐test. (D) SaOS2 (3 × 10^3^, 72 h), U2OS (2 × 10^3^, 72 h), 143B (1 × 10^3^, 72 h), HOS (2 × 10^3^, 72 h), MG63 (2 × 10^3^, 72 h), and LM8 (1 × 10^3^, 72 h) were treated with the FLT‐3 inhibitor at concentrations ranging from 0 to 1000 nm (*N* = 3 per group). The relative cell proliferation rates were determined using the WST‐1 assay. Bars represent the SD. *P < 0.05 and **P < 0.01 in Student’s t‐test. (E) 143B (1 × 10^6^), HOS (1 × 10^6^), MG63 (1 × 10^6^), SaOS2 (2 × 10^6^), U2OS (2 × 10^6^), and LM8 (1 × 10^6^) were treated with TAS‐115 at concentrations ranging from 0 to 10 μm for 48 h. Cell viability was determined using a Countess automated cell counter (Invitrogen). Bars represent the SD. *P < 0.05 and **P < 0.01 in Student’s *t*‐test. (F) Calculated IC_50_ values of each cell line against TAS‐115.

We investigated the phosphorylation of PDGFRα, AXL, and FLT‐3 in not only the murine OS cell line but also several human OS cell lines and examined the effect of PDGFRα, AXL, and FLT‐3 signaling on their proliferation using various selective inhibitors. The phosphorylation of PDGFRα, AXL, and FLT‐3 was observed in almost all cell lines (Fig. 6A), and these inhibitors induced a dose‐dependent growth inhibition in many cell lines (Fig. 6B–D). These results indicate that these signaling pathways were partly involved in the proliferation of OS cells. TAS‐115 treatment blocked the phosphorylation of PDGFRα, AXL, and FLT‐3 and suppressed the proliferation of all OS cell lines in a dose‐dependent manner (Fig. 6E). The 50% inhibitory concentration (IC_50_) values of TAS‐115 were as follows: 143B: 4.00 μmol·L^−1^; HOS: 3.16 μmol·L^−1^; MG63: 1.26 μmol·L^−1^; U2OS: 7.77 μmol·L^−1^; SaOS2: 8.16 μmol·L^−1^; and LM8: 2.55 μmol·L^−1^ (Fig. 6F). These observations indicate that TAS‐115 suppressed the proliferation of OS cells by inhibiting all signaling of PDGFRα, FLT‐3, and AXL.

## Discussion

This study showed three main findings. First, the novel multiple RTK inhibitor TAS‐115 had an antitumor effect on the highly metastatic mouse OS cell line LM8. Second, this antitumor effect of TAS‐115 was partially attributed to the blocking of PDGFRα, AXL, and FLT‐3 phosphorylation. Third, these signals were also associated with the proliferation of many human OS cell lines and TAS‐115 inhibited the proliferation of these human OS cell lines with inhibition of these signals. These results suggested that TAS‐115 could be a particularly potent agent against OS.

Our laboratory previously reported that nuclear factor‐κB (NF‐κB) is a potential molecular target for designing specific prophylactic interventions against distant metastasis [14, 15, 16]. In the past study, an NF‐κB inhibitor did not suppress the growth of the pulmonary metastasis formed once. Interestingly, in this study, we showed that TAS‐115 is able to directly suppress the growth of a formed lung micro‐metastasis in an intravenous injection mouse model. We believe that TAS‐115 may be a more useful therapeutic drug than the NF‐κB inhibitor for patients who have already had pulmonary metastases.

We showed that the antitumor activity of TAS‐115 is partially mediated via PDGFRα/AXL/FLT‐3 signaling, thereby supporting the previous findings of Fujita *et al*. [5], who discovered TAS‐115 and showed that it is capable of inhibiting both MET and VEGFR2 via ATP antagonism. They reported that TAS‐115 inhibits MET, VEGFR2, and several other tyrosine kinases with IC_50_ values of less than 1 μmol·L^−1^, including PDGFRα, AXL, and FLT‐3 in mobility shift assay. We did not detect the phosphorylation of MET and VEGFR2 in the LM8 cell lysate. However, the phosphorylation of PDGFRα, AXL, and FLT‐3 was detected in the LM8 lysate *in vivo*. Furthermore, we showed that TAS‐115 inhibited the phosphorylation of PDGFRα, AXL, and FLT‐3 and decreased cell proliferation in LM8 and other human osteosarcoma cell lines. Thus, our results indicated that these three signals are potential targets of TAS‐115 against osteosarcomas.

We demonstrated that these signals were also activated in various human OS cell lines and that they were associated with their proliferation. Several studies have reported that these signals are associated with OS prognosis. For example, PDGFRα and PDGF‐A correlate with a poor prognosis in OS [17, 18], AXL is frequently overexpressed in OS [19] and promotes osteosarcoma progression by affecting p‐AKT expression [20, 21], and FLT‐3 ligand is significantly associated with poor overall survival and event‐free survival independently of metastasis [22]. However, several clinical studies of selective inhibitors, such as imatinib, demonstrated little or no activity as a single agent in OS [23, 24]. On the other hand, several clinical trials of multiple TK inhibitors, such as regorafenib [25], cabozantinib [26], and apatinib [27], in OS indicated a marked effect for OS and the potential to be developed as new therapeutic agents. TAS‐115 is also a novel multiple RTK inhibitor that demonstrates much less toxicity in various normal cell lines than other VEGFR‐targeted kinase inhibitors. [5]. Currently, a phase 1 study investigating the effect of TAS‐115 in patients with solid tumors, including metastatic and relapsed OS, is ongoing in Japan (JapicCTI‐132333). TAS‐115 may also be a novel treatment for these patients.

We believe that the activated signals of tumor cells are different among tumor microenvironments. Therefore, we investigated the signals of LM8 using phospho‐RTK arrays in three conditions: *in vitro* culture, subcutaneous tumor, and pulmonary metastasis. Interestingly, PDGFRα and AXL were activated in all three conditions. However, FLT‐3 was not activated *in vitro* but was *in vivo*. These observations suggested that tumor cells were affected by the tumor microenvironment. There are few detailed reports about effective multiple TK inhibitors against osteosarcoma and their underlying modes of action. Interestingly, Fioramonti *et al*. [28] reported that cabozantinib, an inhibitor of multiple RTKs such as VEGFR2, AXL, and c‐MET, affects not only tumor cells but also osteoblasts, which induces RANKL expression, promoting tumor proliferation in the primary bone microenvironment. However, the interaction between the tumor and the host microenvironment in lung metastasis (e.g., immune cells and cancer‐associated fibroblasts) is unknown. In our study, the activated signal was different in the subcutaneous tumor compared to the one in the pulmonary metastasis. Therefore, TAS‐115 may exert the same antitumor effect in several tumor microenvironments. Further studies are needed to investigate the effect against tumor and host interactions in lung metastasis.

In conclusion, our findings indicate that TAS‐115 exerts antitumor effects against OS partially via the inhibition of PDGFRα, AXL, and FLT‐3 signaling. This study makes a novel contribution to the literature as it is the first time that TAS‐115 has been studied for its effect on osteosarcoma and metastasis derived from osteosarcoma. We suggest that TAS‐115 could be a promising therapeutic option for patients with metastatic or relapsed OS.

## Conflict of interest

The authors declare no conflict of interest.

## Author contributions

NY, ST, and NN conceived and designed the study; YI, HO, KH, and HY developed the methodology; NY, ST, SN, TN, and SY acquired the data; ST, YI, and HO analyzed and interpreted the data; NY, ST, HO, and NN wrote, reviewed, and/or revised the manuscript; YI, HO, and KH provided administrative, technical, or material support; and HY and NN supervised the study.

## Supporting information


**Fig. S1.** The effect of TAS‐115 on the cell cycle. Cells were treated with 0.1 % DMSO (control) or 0.1–10 μM TAS‐115 for 24 h. After treatment, the cells were stained with PI and analyzed by flow cytometry. (A) MC3T3‐E1. (B) NHDF. (C) Relative G0/G1 cell cycle rates were measured (N=3 per group). Bars represent the SD. ** p < 0.01 in Student’s t‐test.

**Fig. S2.** Body weight of mice that were injected with LM8 cells subcutaneously on the back. Mice were treated with 50 (N=5) or 200 (N=5) mg/kg of TAS‐115, or the vehicle (control, N=4). Bars represent the SE. * p < 0.05 in Student’s t‐test from control.
