# Supplementary figures and images for: TAS‐115 inhibits PDGFRα/AXL/FLT‐3 signaling and suppresses lung metastasis of osteosarcoma
Source: FEBS Open Bio. 2020 Mar 30;10(5):767–79. doi: 10.1002/2211-5463.12827 (PMC7193166; doi:10.1002/2211-5463.12827)

## Supporting Information

Figure S1

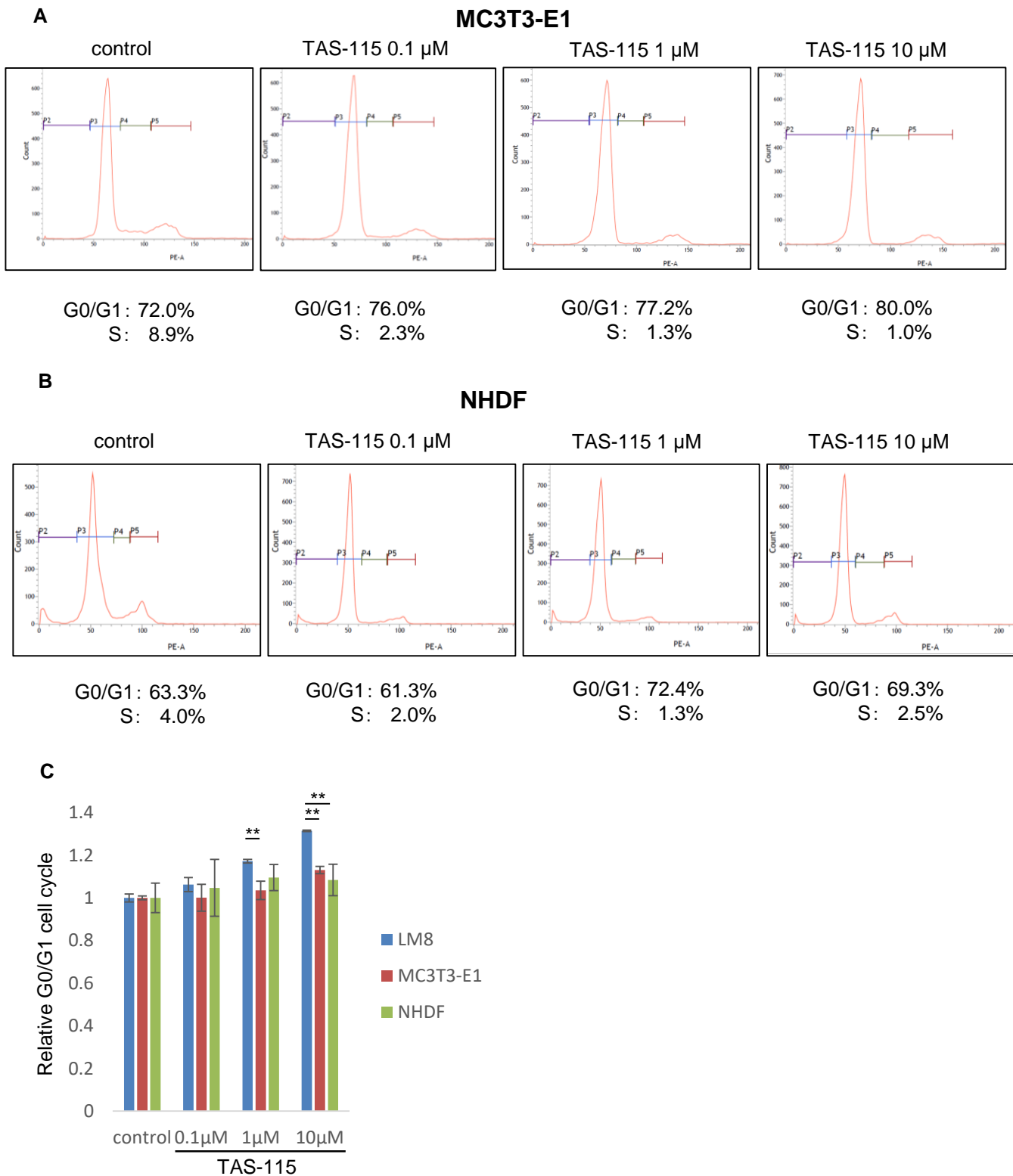

Supplement: Supplementary file 1 — Fig. S1. The effect of TAS‐115 on the cell cycle. Cells were treated with 0.1 % DMSO (control) or 0.1–10 μM TAS‐115 for 24 h. After treatment, the cells were stained with PI and analyzed by flow cytometry. (A) MC3T3‐E1. (B) NHDF. (C) Relative G0/G1 cell cycle rates were measured (N=3 per group). Bars represent the SD. ** p < 0.01 in Student’s t‐test. [file FEB4-10-767-s001.pdf]

**Figure S2**

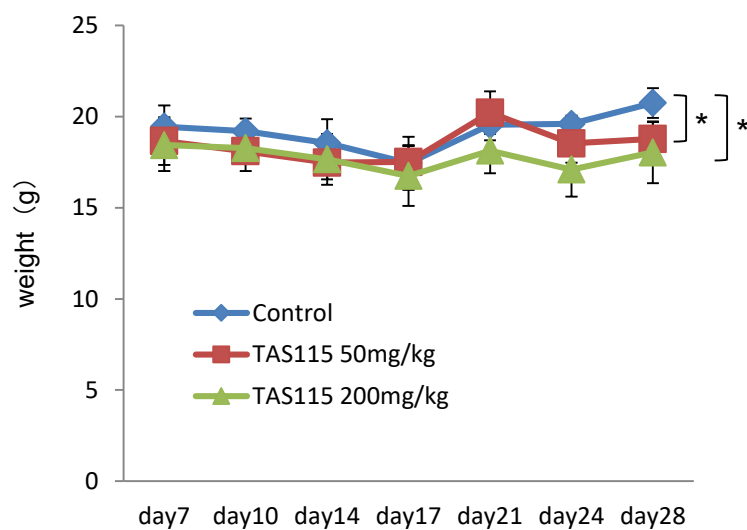

Supplement: Supplementary file 2 — Fig. S2. Body weight of mice that were injected with LM8 cells subcutaneously on the back. Mice were treated with 50 (N=5) or 200 (N=5) mg/kg of TAS‐115, or the vehicle (control, N=4). Bars represent the SE. * p < 0.05 in Student’s t‐test from control. [file FEB4-10-767-s002.pdf]
